# Rapid eye movement sleep behaviour disorder: Past, present, and future

**DOI:** 10.1111/jsr.13612

**Published:** 2022-04-25

**Authors:** Birgit Högl, Isabelle Arnulf, Melanie Bergmann, Matteo Cesari, Ziv Gan‐Or, Anna Heidbreder, Alex Iranzo, Lynne Krohn, Pierre‐Hervé Luppi, Brit Mollenhauer, Federica Provini, Joan Santamaria, Claudia Trenkwalder, Aleksandar Videnovic, Ambra Stefani

**Affiliations:** ^1^ Department of Neurology Innsbruck Medical University Innsbruck Austria; ^2^ Service des Pathologies du Sommeil, Hôpital Pitié‐Salpêtrière Paris France; ^3^ Faculty of Medicine Sorbonne University Paris France; ^4^ Montreal Neurological Institute and Hospital McGill University Montréal Québec Canada; ^5^ Department of Neurology & Neurosurgery McGill University Montréal Québec Canada; ^6^ Department of Human Genetics McGill University Montréal Québec Canada; ^7^ Neurology Service, Hospital Clínic de Barcelona, Institut d'Investigacions Biomèdiques August Pi i Sunyer (IDIBAPS), Centro de Investigación Biomédica en Red sobre Enfermedades Neurodegenerativas (CIBERNED:CB06/05/0018‐ISCIII) Barcelona University of Barcelona Barcelona Spain; ^8^ Centre of Neuroscience of Lyon UMR 5292 CNRS/U1028 INSERM Lyon France; ^9^ Centre Hospitalier Le Vinatier Bron France; ^10^ Paracelsus‐Elena‐Klinik Kassel Germany; ^11^ Department of Neurology University Medical Center Göttingen Göttingen Germany; ^12^ IRCCS Institute of Neurological Sciences UOC NeuroMet, Bellaria Hospital Bologna Italy; ^13^ Department of Biomedical and NeuroMotor Sciences University of Bologna Bologna Italy; ^14^ Department of Neurosurgery University Medical Center Göttingen Germany; ^15^ Department of Neurology, Massachusetts General Hospital Boston Massachusetts USA

**Keywords:** alpha‐synuclein, dream enactment, RBD, REM sleep without atonia, REM‐parasomnia, RWA

## Abstract

This manuscript presents an overview of REM sleep behaviour disorder (RBD) with a special focus on European contributions. After an introduction examining the history of the disorder, we address the pathophysiological and clinical aspects, as well as the diagnostic issues. Further, implications of RBD diagnosis and biomarkers are discussed. Contributions of European researchers to this field are highlighted.

## 1 INTRODUCTION: HISTORY OF RAPID EYE MOVEMENT SLEEP BEHAVIOUR DISORDER (RBD)

The first description of dream‐enacting behaviours dates back to the early Roman empire where reports on animals were provided as proof that non‐human animals also dream (Pline l'Ancien, 1961). In the 17th century, Miguel de Cervantes described an episode of dream‐enacting behaviour (Iranzo et al., 2004), in which Don Quixote shouts and vigorously attacks some wineskins while dreaming that he is fighting a giant. In 1881, Lasègue observed that patients with delirium tremens presented with dream‐like behaviours, vocalisations, and rampaging in bed (Lasègue, 1881), features possibly resembling rapid eye movement (REM) sleep behaviour disorder (RBD). A major advance was made in 1965 when Jouvet and Delorme (1965) created an RBD model in cats 20 years before it was identified as a disorder in humans. Sastre and Jouvet sought to determine the origin of REM sleep muscle atonia and injected ibotenic acid in various nuclei to suppress atonia. They observed that lesions of the perilocus coeruleus alpha (an equivalent to the human locus subcoeruleus) resulted in dream‐like behaviours (“oneiric behaviours”), including predatory attack, rage, flight, and grooming in cats (Sastre & Jouvet, 1979).

Europeans were also among the first to observe REM sleep without atonia (RWA) in individuals with parkinsonism. In 1969, Traczynska‐Kubin et al. (1969) observed patients with Parkinson's disease (PD) having persistent mental electromyography (EMG) activity despite REMs resembling REM sleep. In 1975, Mouret (1975) described a persistence of mental EMG activity during paradoxical sleep in patients with PD compared with healthy individuals. Similar findings were named “stage 1‐REM” by Japanese researchers (for a review see Tachibana, 2009) and “stage 7” sleep by Guilleminault et al. (1976) in patients with narcolepsy treated with clomipramine. The Europeans were also the first to document video‐polysomnographic (v‐PSG) recording of an RBD episode in a patient with Shy‐Drager syndrome (Coccagna et al., 1985). Shortly after, Salva and Guilleminault (1986) described the disappearance of REM sleep atonia and the appearance of complex dream‐enacting behaviours in patients with olivopontocerebellar degeneration. Following on from this there were several reports by Japanese researchers of stage 1‐REM, possibly resembling RWA in otherwise healthy‐seeming elderly individuals experiencing their first manifestations of somnambulism‐like behaviours (Tachibana et al., 1991), as well as in patients with brainstem degeneration (Shimizu et al., 1990), and under clomipramine treatment (Niiyama et al., 1993). A more detailed history of the detection of RWA and RBD has been provided in a review (Frauscher & Högl, 2012).

The landmark discovery of RBD as a disorder is attributed to Carlos Schenck and Mark Mahowald who described the first case series of people showing dream‐enacting behaviours linked to a polysomnographic confirmation of RWA and gave RBD its name. Schenck and Mahowald identified the sleep‐related violence and risk of injury, linked it with Jouvet's cat model, and discovered the benefit of clonazepam (Schenck et al., 1986). In the first brain neuropathological examination of a case with isolated RBD (iRBD), abnormal α‐synuclein deposits were found in the locus coeruleus/subcoeruleus (Uchiyama et al., 1995). Schenck and Mahowald followed up their first 30 idiopathic RBD patients finding that one‐third of their cohort later converted to parkinsonism (Schenck et al., 1996). This major finding occurred before Braak et al. suggested that abnormal α‐synuclein propagates in a bottom‐up pattern in prodromal and defined PD, starting in the gut and medulla oblongata (stage 1), then in the locus coeruleus/subcoeruleus (stage 2) and later in the substantia nigra (stages 3 and 4) and cortex (stages 5 and 6) (Del Tredici et al., 2002). In 2007, Boeve, Silber, et al. (2007b) postulated that dysfunction in the sublaterodorsal nucleus (corresponding to stage 2 in the Braak staging system) could lead to RWA and RBD. This phenoconversion from RBD to parkinsonism and dementia was later confirmed in several countries, and other synucleinopathies were associated with RBD (Fantini et al., 2005; Iranzo et al., 2006).

### 1.1 RBDpathophysiology

Glutamatergic neurons located in a small pontine nucleus localised ventral to the laterodorsal tegmental nucleus (LDT) – named sublaterodorsal tegmental nucleus (SLD) in rats and locus subcoeruleus in humans – are responsible for inducing muscle atonia during REM sleep. These neurons express the vesicular transporter 2 of glutamate (vGLUT2), the specific marker of glutamatergic neurons, and cFos, a marker of activation in rats displaying a REM sleep hypersomnia (Clement et al., 2011). Unit recordings of these neurons confirmed that they are selectively active during REM sleep (Boucetta et al., 2014). It has also been shown that genetic inactivation of glutamatergic SLD neurons in rats and mice induces RBD and a 30% decrease in REM sleep quantities (Uchida et al., 2021; Valencia Garcia et al., 2017).

It has also been shown that combined microdialysis of bicuculline (a GABA‐A antagonist), strychnine (glycine antagonist), and phaclophen (a GABA‐B antagonist) in the trigeminal nucleus are necessary to restore jaw muscle tone during REM sleep (Brooks & Peever, 2012). Furthermore, SLD neurons directly excite GABA/glycinergic neurons located in the ventral medullary reticular nuclei (Boissard et al., 2002; Valencia Garcia et al., 2017; Valencia Garcia et al., 2018). Nearly all c‐Fos‐labelled neurons localised in these nuclei express GAD67 and glycine transporter 2mRNA after 3 h of paradoxical sleep recovery following 72 h of paradoxical sleep deprivation (Sapin et al., 2009; Valencia Garcia et al., 2018). These neurons directly project to spinal motoneurons (Valencia Garcia et al., 2018). Inactivation of the GABA and glycinergic neurons of these nuclei in rats and mice induces RBD (Uchida et al., 2021; Valencia Garcia et al., 2018). All these experiments indicate that GABA/glycine neurons located in the ventral medulla project to and hyperpolarise motoneurons during REM sleep leading to muscle atonia.

Interestingly, functional neuroimaging and postmortem brain studies report the presence of Lewy bodies and neuronal loss in SLD and ventral medulla (Arnulf, 2012; Boeve, Dickson, et al., 2007a; Iranzo et al., 2013). It is, therefore, probable that RBD is due to specific neurodegeneration of the glutamate SLD and/or GABA/glycine medullary neurons.

It is well accepted that motoneurons are also phasically excited by glutamate during REM sleep (Burgess et al., 2008). It is, therefore, likely that RBD behaviours are due to phasic excitation of motoneurons by glutamate in the absence of tonic GABA/glycine inhibition. Glutamate pre‐motoneurons are interneurons located in close vicinity of motoneurons and neurons located in ponto‐medullary reticular nuclei and the red nucleus (Rekling et al., 2000). These neurons are directly excited by glutamatergic pyramidal neurons of the motor cortex to induce voluntary movements (Rekling et al., 2000). The reticular formation and the red nucleus could play a major role in exciting motoneurons during REM sleep without the need for cortical activation (Blumberg & Plumeau, 2016; Del Rio‐Bermudez et al., 2015). However, patients often show long and complex behaviours, such as singing and giving long speeches, which strongly suggests that the motor cortex drives such behaviours (De Cock et al., 2007). In line with this, activation of the human motor cortex, similar to that observed during a voluntary movement during wakefulness, has been observed during REM sleep (De Carli et al., 2016).

Due to the characteristic emotional component of RBD dream‐enacting behaviours and vocalisations, involvement of the limbic system has been postulated. This hypothesis is supported by animal studies on cats and by reports of RBD in humans affected by limbic encephalitis (Iranzo, 2018).

Everything considered, RBD is likely induced by neurodegeneration of the ponto‐medullary GABA/glycinergic and/or glutamatergic neuronal system physiologically inducing muscle atonia during REM sleep, although other circuits are also involved in the generation of RWA and dream‐enacting behaviours.

### 1.2 Clinical features of isolatedRBD(iRBD)

The prevalence of iRBD in the general population over 60 years old is 0.5–1% (Haba‐Rubio et al., 2018; Pujol et al., 2017), and the mean age at clinical consultation is usually in the seventh decade. iRBD is rare in those under 50 years (Fernandez‐Arcos et al., 2016). For unknown reasons, people seeking medical consultation for iRBD at sleep centres are more frequently men than women, although this finding may be due to a selection bias as RBD in men is more violent than in women. Ageing, head injury, farming, pesticide exposure, antidepressant therapy, and GBA mutations are all risk factors for developing iRBD (Postuma et al., 2012). Hyposmia, depression, and constipation are more common in iRBD than in controls (Aguirre‐Mardones et al., 2015).

The main clinical features of iRBD are dream‐enacting behaviours and nightmares, but this symptomatology also occurs in posttraumatic sleep disorder, sleep terrors, as well as in some patients with severe obstructive sleep apnoea and periodic limb movement disorder. Recalled dreams are short, vivid, intense, frightening, and negatively toned (e.g., being attacked or chased by an unknown person for an unknown reason). Clinical manifestations consist in vocalisations (e.g., yelling, swearing, crying, or laughing) and vigorous behaviours (punching, kicking, jumping out of bed) where patients appear to be enacting their dreams. These behaviours occur in REM sleep, with eyes closed, are usually confined to the bed, and may result in injuries to the patient and the bed partner (Fernandez‐Arcos et al., 2016). However, the most common abnormal behaviours seen in REM sleep during video‐polysomnography (v‐PSG) are prominent jerks. Interestingly, an important proportion of iRBD patients report good sleep quality and are unaware of their nocturnal episodes, indicating that bed partners are essential for informing about and describing these behaviours. To reduce the intensity and frequency of the nightmares and motor behaviours (therefore, also reducing the risk of injury) clonazepam and melatonin can be used. Both these treatments are suggested as Level B treatment for RBD by the Standards of Practice Committee of the American Academy of Sleep Medicine (Aurora et al., 2010). Clonazepam is usually effective at a dose <2 mg, melatonin at a dose 3–12 mg. Long‐term treatment is usually required. Melatonin has a favourable side effect profile (dose‐related side effects include morning headache, morning sleepiness, and delusions/hallucinations), whereas clonazepam should be used with caution in patients with dementia, gait disorders, or concomitant obstructive sleep apnea. The most common side effects of clonazepam are sedation, impotence, early morning motor incoordination, confusion, and memory dysfunction (Aurora et al., 2010). Besides symptomatic treatment, improving safety within the bedroom environment is also often necessary (Fernandez‐Arcos et al., 2016).

After 15 years of follow‐up, about 95% of iRBD patients will be clinically diagnosed with the synucleinopathies dementia with Lewy bodies (DLB, 45%), PD (45%), or multiple system atrophy (MSA, 5%) (Iranzo et al., 2016). Indeed, the presence of abnormal synuclein in the cerebral spinal fluid (CSF), olfactory mucosa, and peripheral organs (colon, skin, and salivary glands) is detected in most iRBD patients. Markers of short‐term conversion to a clinically overt synucleinopathy are hyposmia, abnormal DAT‐SPECT, mild cognitive impairment, and minor parkinsonian signs. Patients with long‐standing iRBD of more than 15 years may show synuclein in the CSF and organs, hyposmia and DAT deficit, indicating a slow but ongoing neurogenerative process (Hogl et al., 2018; Iranzo et al., 2017; Teigen et al., 2021).

## 2 VIDEO‐POLYSOMNOGRAPHY (V‐PSG) AS A DIAGNOSTIC REQUIREMENT AND PROGRESSION MARKER INRBD

The current criteria for the diagnosis of RBD (American Academy of Sleep Medicine, 2014) require the demonstration of RWA on v‐PSG. Several manual/visual methods have been proposed to quantify RWA, but the most validated method that is also recommended by recent international guidelines (Cesari et al., 2021) is the one proposed by the Sleep Innsbruck Barcelona (SINBAR) group (Frauscher et al., 2012). This method measures “any” (i.e. phasic or tonic) muscular activity in the chin and phasic muscular activity in both flexor digitorum superficialis muscles in the upper limbs, either in 30 s epochs or in 3 s epochs, and the respective cut‐offs of 27% and 32% have shown to be sensitive and specific to distinguish RBD from controls (Frauscher et al., 2012). Quantification of RWA in the lower extremities has lower specificity than in the upper extremities (Cesari et al., 2021). Because manual/visual RWA quantification is extremely laborious, several (semi‐)automatic methods have been proposed. Of these, the REM atonia index (Ferri et al., 2010) is the most validated one, but it quantifies RWA only in the chin. Only one semi‐automatic method measures muscular activity both in the chin and the upper extremities (Frauscher et al., 2014).

RWA is not only the electrophysiological hallmark of RBD but is also a biomarker of neurodegeneration in iRBD (McCarter et al., 2019; Nepozitek et al., 2019) and could potentially be used as a biomarker for impending progression to an overt α‐synucleinopathy in iRBD patients, although more studies are needed to confirm this.

v‐PSG documentation of behaviours during REM sleep is not strictly required in the current American Academy of Sleep Medicine (AASM) criteria (American Academy of Sleep Medicine, 2014), but new diagnostic guidelines by the International RBD study group (Cesari et al., 2021) require the demonstration of at least one RBD episode. An RBD episode is defined as one or more motor events and/or vocalisation in REM sleep suggestive of dream enactment, thus including jerky, discontinuous simple and complex movements with or without vocalisations. Studies have shown that large, vigorous movements during REM sleep represent only the tip of the iceberg in RBD patients, who mostly have simple, minor jerks, requiring a careful video analysis (Frauscher et al., 2007). Research to develop automatic tools that can identify such movements and, therefore, help to diagnose RBD (Waser et al., 2020), is essential.

### 2.1 Sex issues inRBD

Since the seminal first description of five patients with RBD, a male predominance has been reported (Schenck et al., 1986). This was supported by subsequent studies that reported up to 80% of RBD patients being male (Schenck et al., 2019).

A more recent epidemiological study, however, suggested that the prevalence of RWA does not differ between the sexes (Haba‐Rubio et al., 2018). As women with RBD often present less violent dreams and behaviours, it has been suggested that RBD might be underestimated in women due to milder symptoms or lack of perception by bed partners (Fernandez‐Arcos et al., 2016).

Differences in dream content between men and women, investigated in people with PD‐RBD, showed that women dream more about activities of daily living, family and friends, while men have more performance‐related dreams, including about sports, employment and can also be more aggressive (Borek et al., 2007), although data on this in iRBD are lacking. Nonetheless, differences in dream content could explain why men more often show harmful behaviours during RBD episodes (Mahale et al., 2016) and have a higher risk of injuries compared with women (Comella et al., 1998).

In line with this, a polysomnographic study investigating motor features of sleep behaviours reported that men have higher EMG phasic activity, more myoclonic movements, and more movements involving the trunk, whereas segmental movements were more frequent in women (Bugalho & Salavisa, 2019). Another polysomnographic study investigating RWA in both legs and arms, reported a higher RWA index in the legs in men compared with women, and a higher RWA index in the arms in women. The authors postulated that this finding might reflect a different character of RWA among the sexes (Borek et al., 2007; Tatman & Sind, 1996).

Despite frequent reports of sex differences in RBD phenotypes, this aspect has yet to be studied extensively. Future studies are needed to better elucidate the reasons for sex differences in the prevalence of RBD, as well as to characterise potential differences in muscle activity and motor events during REM sleep between women and men (Bodkin & Schenck, 2009; Borek et al., 2007; Bugalho & Salavisa, 2019; Comella et al., 1998; Fernandez‐Arcos et al., 2016; Haba‐Rubio et al., 2018; Mahale et al., 2016; Schenck et al., 1986; Tatman & Sind, 1996; Zhou et al., 2015).

### 2.2 The genetics ofRBD

Great strides forward have been made in iRBD genetic research over the past several years. iRBD likely has a distinct genetic risk profile compared with overt α‐synucleinopathies, with evidence of both overlapping and contrasting risk loci across the conditions. RBD heritability, explained by common variants, is estimated at 12.3%, which is similar to the current estimation for DLB, and five RBD genetic loci have been identified by a genome‐wide association study (GWAS) (Krohn et al., 2021). These genes are concentrated in the autophagy‐lysosomal pathway (ALP) and are more specific to this pathway than any similarly powered GWAS of PD or DLB. Each of these RBD genes, *SNCA*, *GBA*, *TMEM175*, *INPP5F*, and *SCARB2*, are nominated PD risk loci (Nalls et al., 2019). However, the driving risk variants in *SNCA* and *SCARB2* are independent, meaning different genetic mechanisms are driving the risk for RBD and PD at these loci. The top *SNCA* variant associated with an increased risk for PD may have an opposite effect in RBD, decreasing the risk (Krohn et al., 2020). There is evidence that these RBD and PD variants may be affecting gene expression differently, and in different brain regions, with RBD variants localised in cortical regions (Krohn et al., 2021). Additionally, prominent PD genes such as *LRRK2* (Fernandez‐Santiago et al., 2016), *MAPT*, and autosomal recessive genes (Mufti, Rudakou, et al., 2021a) are not associated with isolated RBD. A similar pattern is found between RBD and DLB, where ALP genes *SNCA*, *GBA*, and *TMEM175* are shared risk factors in both conditions, however, DLB genes *APOE* and *BIN1* (Chia et al., 2021) are not associated with RBD (Gan‐Or et al., 2017; Krohn et al., 2021). Mutations in *PSAP*, encoding for saposin C, a lysosomal activator of *GBA*, have also been implicated in iRBD (Sosero et al., 2022), as well as rare variants in *LAMP3* (encoding the lysosomal associated membrane protein 3) and other genes (Mufti, Yu, et al., 2021b). Overall, the shared loci across RBD and overt α‐synucleinopathies are localised in the ALP, and genes associated with other neurodegenerative mechanisms (e.g., tau aggregation, mitochondrial dysfunction) do not appear to play a major role in RBD susceptibility. Genetics may also contribute to the phenoconversion rate from RBD to overt neurodegeneration; studies in *SNCA* (Krohn et al., 2020) and *GBA* (Honeycutt et al., 2019) show evidence that risk variants are associated with rapid phenoconversion, however, with limited confidence at these sample sizes.

### 2.3 Biomarkers – classical and recent

Clinical trials with putative neuropreventive strategies in PD have been largely unsuccessful in the past, in part because the target populations were early/de novo PD patients with characteristic motor symptoms already present. At this stage, the degeneration of dopaminergic neurons is advanced with over 50% of nigrostriatal dopaminergic neurons already being affected by neurodegeneration. Therefore, strategies to prevent future disease conversion need to focus on risk cohorts. To achieve this important step, studies should be conducted in iRBD patients to assess progression biomarkers in the early disease stage in parallel to larger cohorts of at‐risk individuals. With an average of only 6.3% of iRBD patients converting to disease per year (Postuma et al., 2019), conversion alone cannot serve as an outcome measure for future clinical trials and further objective trait markers are needed. Also, individuals with iRBD convert into different α‐synuclein aggregation diseases (i.e., PD, MSA, DLB). It may, therefore, be important to stratify these groups in the prodromal state. For prodromal individuals and population‐based screens, which are becoming more popular for identifying individuals at risk, we need biomarkers that are less invasive than CSF, as well as widely applicable screening instruments. Several strategies are currently being investigated, including imaging studies (e.g., dopamine transporter imaging, ^18^F FDG‐PET), objective quantitative assessment of emerging mild motor disease, cognitive testing and tissue analyses with aggregation chemistry for α‐synuclein (Miglis et al., 2021).

The total α‐synuclein in CSF is 15% lower in PD and MSA, while in iRBD levels are slightly higher for, as yet, unknown reasons (Mollenhauer et al., 2019). More promising is the detection of α‐synuclein aggregation by seeding aggregation assays (SAA; RT‐QuiC or PMCA) with sensitivities and specificities above 90–95% for PD in CSF (Shahnawaz et al., 2017). One study in iRBD showed a sensitivity of 90.4% and a specificity of 90.0%. In one individual, positivity of SAA was detected 10 years before the conversion to disease (Iranzo et al., 2021) Major hurdles faced by SAA are the lack of quantification and the fact that the best performance is shown in CSF compared with colon, salivary glands, olfactory mucosa, and skin (Antelmi et al., 2017; Doppler et al., 2017; Fernandez‐Arcos et al., 2016; Iranzo et al., 2018; Sprenger et al., 2015; Stefani & Hogl, 2021; Vilas et al., 2016). The current mechanism of the molecular processes leading to α‐synuclein aggregation and disease are still unknown and cannot yet be explained in relation to the assay parameters. While for CSF the results are promising, the first studies also report SAA positivity in skin samples in PD as well as in olfactory mucosa (De Luca et al., 2019; Wang et al., 2020). Some groups also show different seeding dynamics in patients with MSA and PD, which could indicate different α‐synuclein strains (Shahnawaz et al., 2020).

A more peripherally acting biomarker is the neurofilament light chain (NfL), which can now reliably be quantified in peripheral blood. Although nonspecific and elevated in several other neurological diseases, slightly higher values of plasma NfL are shown for PD and RBD and markedly higher for MSA patients (Mollenhauer et al., 2020).

In the future, we will know if other biomarkers such as inflammatory panels, faecal microbiome, and miRNA hold their promise of being adequate screening tools for those at risk of converting to disease. Unfortunately, none of the previous biomarkers, including SAA, can be currently used for individual quantification to differentiate between individuals at risk for conversion and those remaining free of a neurodegenerative disease. Larger cohorts are needed.

### 2.4 Implications of theRBDdiagnosis

The implications of establishing the diagnosis of RBD are multifaceted. These encompass (i) clinical implications centred on the management strategies and risk mitigation of potential injurious RBD behaviours, (ii) implications related to very strong associations between RBD and evolving synuclein‐specific neurodegeneration, (iii) the need to increase awareness about this disorder among physicians, allied health professionals, and the general public, and (iv) the opportunity to engage numerous stakeholders around RBD as a unique disorder that is positioned on the intersection of neurology, sleep medicine, and neuroscience.

The clinical implications of RBD as a parasomnia are centred on symptomatic management and safety precautions in sleeping environments aimed at preventing injuries from RBD‐related motor behaviours (St Louis & Boeve, 2017). The relative paucity of well‐designed randomised symptomatic clinical trials should be a call for action for a more robust clinical trial RBD pipeline (Gilat et al., 2020; Shin et al., 2019). The success of such trials will largely depend on the adequate selection of outcome measures and trial duration, which will allow for proper ascertainment of the intervention's effectiveness.

Implications of RBD as a prodromal stage of an evolving α‐synucleinopathy are complex. RBD patients are increasingly becoming aware of their risk of developing one of these disorders in the future. This necessitates proper counselling on the risk of phenoconversion so that appropriate planning for the future can be considered. Currently, there is a substantial gap in prognostic counselling offered to the patient as reports indicate that only 50% of patients with RBD receive counselling (Feinstein et al., 2019). There is, therefore, a need to develop best practices for prognostic counselling in iRBD.

One of the most important implications of RBD as a prodromal synucleinopathy is an opportunity to position the iRBD population as an ideal study cohort for testing disease‐modifying treatments aimed at delaying or preventing phenoconversion to a synucleinopathy (Hogl et al., 2018). Selecting the ideal patient candidates for disease‐modifying trials, having effective and reliable screening and recruitment methods, employing robust clinical trial designs, and choosing appropriate outcome measures capable of demonstrating disease modification within the reasonable trial duration are the critical aspects of trial planning for iRBD. Some of these elements are more straightforward than others. The International RBD study group published two consensus statements on clinical trials in the RBD population that detail these aspects relevant to organisations and implementation of RBD clinical trials (Schenck et al., 2013; Videnovic et al., 2020).

Finally, raising awareness and promoting education about RBD is a very important implication of RBD, which is overall underdiagnosed, or the diagnosis is quite delayed. Bringing together various stakeholders such as clinicians, scientists, patients, disease‐specific foundations, government agencies, industry, and the general public will enable us to advance various aspects of RBD clinic care, research, and therapeutic development.

## 3 FUTURE DIRECTIONS

Since the first description of RBD, the disease has been better characterised through pathophysiologic, clinical and v‐PSG studies, as well as through extensive research on biomarkers and genetics.

Despite these advances, the phase of phenoconversion from iRBD to overt α‐synucleinopathy still needs to be further investigated. A combination of biomarkers will likely allow better identification of those with iRBD at high risk of short‐term phenoconversion, whereas it is still unclear how iRBD individuals that go on to develop PD will be differentiated from those phenoconverting into DLB or MSA. Although a combination of biomarkers might be useful, it is also likely that a better phenotypic characterisation will be achieved through the identification of biomarkers or α‐synuclein aggregates in biofluids or tissues, which are specific for one α‐synucleinopathy (i.e., DLB, PD or MSA).

Another aspect that will become more relevant in the future and deserves further investigation is prodromal RBD. It has been defined as a stage in which symptoms and signs of evolving RBD are present, but do not yet meet established diagnostic criteria (Cesari et al., 2021; Hogl et al., 2018). International guidelines for the identification of prodromal RBD have been published recently by the International RBD study group, providing a framework that will ensure harmonised studies and, therefore, a better understanding of the clinical relevance of this condition and its evolution into iRBD.

All the previously mentioned aspects, as well as the identification of RBD in the general population, will likely take advantage of the implementation of artificial intelligence. Machine‐learning approaches may provide new ways of identifying RBD patients (e.g., considering both muscle activity and movements), as well as new ways of phenotypic characterisation (e.g., based on a combination of biomarkers), dramatically improving detection and classification of RBD.

Some open issues in the RBD research field concern treatment. New insights into pathophysiology deriving from basic science may lead to the development of novel treatments acting on specific pathways involved in the generation of RBD. Double‐blind, randomised, controlled trials are still needed to assess the efficacy of symptomatic RBD treatment. Moreover, clinical trials testing neuroprotective drugs may represent a turning point in the development of disease‐modulating treatments for alpha‐synucleinopathies.

## AUTHORS' CONTRIBUTIONS

BH and AS planned, coordinated, and edited the manuscript. All authors drafted a section of the work and revised the entire work critically for important intellectual content and gave final approval of the version to be published.

## CONFLICT OF INTEREST

Author conflicts of interest in relation to the content of this manuscript.

## Data Availability

Data sharing is not applicable to this article as no new data were created or analyzed in this study.

## References

[jsr13612-bib-0001] Aguirre‐Mardones, C. , Iranzo, A. , Vilas, D. , Serradell, M. , Gaig, C. , Santamaria, J. , & Tolosa, E. (2015). Prevalence and timeline of nonmotor symptoms in idiopathic rapid eye movement sleep behavior disorder. Journal of Neurology, 262(6), 1568–1578. 10.1007/s00415-015-7742-3 25929658

[jsr13612-bib-0002] American Academy of Sleep Medicine (2014). In I. L. Darien (Ed.), The international classification of sleep disorders: Diagnostic and coding manual (3rd ed., Rev. edn). American Academy of Sleep Medicine.

[jsr13612-bib-0003] Antelmi, E. , Donadio, V. , Incensi, A. , Plazzi, G. , & Liguori, R. (2017). Skin nerve phosphorylated alpha‐synuclein deposits in idiopathic REM sleep behavior disorder. Neurology, 88(22), 2128–2131. 10.1212/WNL.0000000000003989 28468843

[jsr13612-bib-0004] Arnulf, I. (2012). REM sleep behavior disorder: Motor manifestations and pathophysiology. Movement Disorders, 27(6), 677–689. 10.1002/mds.24957 22447623

[jsr13612-bib-0005] Aurora, R. N. , Zak, R. S. , Maganti, R. K. , Auerbach, S. H. , Casey, K. R. , Chowdhuri, S. , Karippot, A. , Ramar, K. , Kristo, D. A. , Morgenthaler, T. I. , Standards of Practice Committee , & American Academy of Sleep Medicine . (2010). Best practice guide for the treatment of REM sleep behavior disorder (RBD). Journal of Clinical Sleep Medicine, 6(1), 85–95. 20191945 PMC2823283

[jsr13612-bib-0006] Blumberg, M. S. , & Plumeau, A. M. (2016). A new view of "dream enactment" in REM sleep behavior disorder. Sleep Medicine Reviews, 30, 34–42. 10.1016/j.smrv.2015.12.002 26802823 PMC4912466

[jsr13612-bib-0007] Bodkin, C. L. , & Schenck, C. H. (2009). Rapid eye movement sleep behavior disorder in women: Relevance to general and specialty medical practice. Journal of Women's Health (2002), 18(12), 1955–1963. 10.1089/jwh.2008.1348 20044857

[jsr13612-bib-0008] Boeve, B. F. , Dickson, D. W. , Olson, E. J. , Shepard, J. W. , Silber, M. H. , Ferman, T. J. , Ahlskog, J. E. , & Benarroch, E. E. (2007a). Insights into REM sleep behavior disorder pathophysiology in brainstem‐predominant Lewy body disease. Sleep Medicine, 8(1), 60–64. 10.1016/j.sleep.2006.08.017 17157062 PMC2702126

[jsr13612-bib-0009] Boeve, B. F. , Silber, M. H. , Saper, C. B. , Ferman, T. J. , Dickson, D. W. , Parisi, J. E. , Benarroch, E. E. , Ahlskog, J. E. , Smith, G. E. , Caselli, R. C. , Tippman‐Peikert, M. , Olson, E. J. , Lin, S. C. , Young, T. , Wszolek, Z. , Schenck, C. H. , Mahowald, M. W. , Castillo, P. R. , Del Tredici, K. , & Braak, H. (2007b). Pathophysiology of REM sleep behaviour disorder and relevance to neurodegenerative disease. Brain, 130(Pt 11), 2770–2788. 10.1093/brain/awm056 17412731

[jsr13612-bib-0010] Boissard, R. , Gervasoni, D. , Schmidt, M. H. , Barbagli, B. , Fort, P. , & Luppi, P. H. (2002). The rat ponto‐medullary network responsible for paradoxical sleep onset and maintenance: A combined microinjection and functional neuroanatomical study. The European Journal of Neuroscience, 16(10), 1959–1973. 10.1046/j.1460-9568.2002.02257.x 12453060

[jsr13612-bib-0011] Borek, L. L. , Kohn, R. , & Friedman, J. H. (2007). Phenomenology of dreams in Parkinson's disease. Movement Disorders, 22(2), 198–202. 10.1002/mds.21255 17133561

[jsr13612-bib-0012] Boucetta, S. , Cisse, Y. , Mainville, L. , Morales, M. , & Jones, B. E. (2014). Discharge profiles across the sleep‐waking cycle of identified cholinergic, GABAergic, and glutamatergic neurons in the pontomesencephalic tegmentum of the rat. The Journal of Neuroscience, 34(13), 4708–4727. 10.1523/JNEUROSCI.2617-13.2014 24672016 PMC3965793

[jsr13612-bib-0013] Brooks, P. L. , & Peever, J. H. (2012). Identification of the transmitter and receptor mechanisms responsible for REM sleep paralysis. The Journal of Neuroscience, 32(29), 9785–9795. 10.1523/JNEUROSCI.0482-12.2012 22815493 PMC6621291

[jsr13612-bib-0014] Bugalho, P. , & Salavisa, M. (2019). Factors influencing the presentation of REM sleep behavior disorder: The relative importance of sex, associated neurological disorder, and context of referral to polysomnography. Journal of Clinical Sleep Medicine, 15(12), 1789–1798. 10.5664/jcsm.8086 31855164 PMC7099179

[jsr13612-bib-0015] Burgess, C. , Lai, D. , Siegel, J. , & Peever, J. (2008). An endogenous glutamatergic drive onto somatic motoneurons contributes to the stereotypical pattern of muscle tone across the sleep‐wake cycle. The Journal of Neuroscience, 28(18), 4649–4660. 10.1523/JNEUROSCI.0334-08.2008 18448642 PMC6670438

[jsr13612-bib-0016] Cesari, M. , Heidbreder, A. , St Louis, E. K. , Sixel‐Doring, F. , Bliwise, D. L. , Baldelli, L. , Bes, F. , Fantini, M. L. , Iranzo, A. , Knudsen‐Heier, S. , Mayer, G. , McCarter, S. , Nepozitek, J. , Pavlova, M. , Provini, F. , Santamaria, J. , Sunwoo, J. S. , Videnovic, A. , Högl, B. , … Stefani, A . (2021). Video‐polysomnography procedures for diagnosis of rapid eye movement sleep behavior disorder (RBD) and the identification of its prodromal stages: Guidelines from the international RBD study group. Sleep, 45(3):zsab257. 10.1093/sleep/zsab257 34694408

[jsr13612-bib-0017] Chia, R. , Sabir, M. S. , Bandres‐Ciga, S. , Saez‐Atienzar, S. , Reynolds, R. H. , Gustavsson, E. , Walton, R. L. , Ahmed, S. , Viollet, C. , Ding, J. , Makarious, M. B. , Diez‐Fairen, M. , Portley, M. K. , Shah, Z. , Abramzon, Y. , Hernandez, D. G. , Blauwendraat, C. , Stone, D. J. , Eicher, J. , … Scholz, S. W. (2021). Genome sequencing analysis identifies new loci associated with Lewy body dementia and provides insights into its genetic architecture. Nature Genetics, 53(3), 294–303. 10.1038/s41588-021-00785-3 33589841 PMC7946812

[jsr13612-bib-0018] Clement, O. , Sapin, E. , Berod, A. , Fort, P. , & Luppi, P. H. (2011). Evidence that neurons of the sublaterodorsal tegmental nucleus triggering paradoxical (REM) sleep are glutamatergic. Sleep, 34(4), 419–423. 10.1093/sleep/34.4.419 21461384 PMC3064553

[jsr13612-bib-0019] Coccagna, G. , Martinelli, P. , Zucconi, M. , Cirignotta, F. , & Ambrosetto, G. (1985). Sleep‐related respiratory and haemodynamic changes in Shy‐Drager syndrome: A case report. Journal of Neurology, 232(5), 310–313. 10.1007/BF00313872 3903059

[jsr13612-bib-0020] Comella, C. L. , Nardine, T. M. , Diederich, N. J. , & Stebbins, G. T. (1998). Sleep‐related violence, injury, and REM sleep behavior disorder in Parkinson's disease. Neurology, 51(2), 526–529. 9710029 10.1212/wnl.51.2.526

[jsr13612-bib-0021] De Carli, F. , Proserpio, P. , Morrone, E. , Sartori, I. , Ferrara, M. , Gibbs, S. A. , De Gennaro, L. , Lo Russo, G. , & Nobili, L. (2016). Activation of the motor cortex during phasic rapid eye movement sleep. Annals of Neurology, 79(2), 326–330. 10.1002/ana.24556 26575212 PMC5066659

[jsr13612-bib-0022] De Cock, V. C. , Vidailhet, M. , Leu, S. , Texeira, A. , Apartis, E. , Elbaz, A. , Roze, E. , Willer, J. C. , Derenne, J. P. , Agid, Y. , & Arnulf, I. (2007). Restoration of normal motor control in Parkinson's disease during REM sleep. Brain, 130(Pt 2), 450–456. 10.1093/brain/awl363 17235126

[jsr13612-bib-0023] De Luca, C. M. G. , Elia, A. E. , Portaleone, S. M. , Cazzaniga, F. A. , Rossi, M. , Bistaffa, E. , De Cecco, E. , Narkiewicz, J. , Salzano, G. , Carletta, O. , Romito, L. , Devigili, G. , Soliveri, P. , Tiraboschi, P. , Legname, G. , Tagliavini, F. , Eleopra, R. , Giaccone, G. , & Moda, F. (2019). Efficient RT‐QuIC seeding activity for alpha‐synuclein in olfactory mucosa samples of patients with Parkinson's disease and multiple system atrophy. Translational Neurodegeneration, 8, 24. 10.1186/s40035-019-0164-x 31406572 PMC6686411

[jsr13612-bib-0024] Del Rio‐Bermudez, C. , Sokoloff, G. , & Blumberg, M. S. (2015). Sensorimotor processing in the newborn rat red nucleus during active sleep. The Journal of Neuroscience, 35(21), 8322–8332. 10.1523/JNEUROSCI.0564-15.2015 26019345 PMC4444549

[jsr13612-bib-0025] Del Tredici, K. , Rub, U. , De Vos, R. A. , Bohl, J. R. , & Braak, H. (2002). Where does parkinson disease pathology begin in the brain? Journal of Neuropathology and Experimental Neurology, 61(5), 413–426. 10.1093/jnen/61.5.413 12030260

[jsr13612-bib-0026] Doppler, K. , Jentschke, H. M. , Schulmeyer, L. , Vadasz, D. , Janzen, A. , Luster, M. , Hoffken, H. , Mayer, G. , Brumberg, J. , Booij, J. , Musacchio, T. , Klebe, S. , Sittig‐Wiegand, E. , Volkmann, J. , Sommer, C. , & Oertel, W. H. (2017). Dermal phospho‐alpha‐synuclein deposits confirm REM sleep behaviour disorder as prodromal Parkinson's disease. Acta Neuropathologica, 133, 535–545. 10.1007/s00401-017-1684-z 28180961 PMC5348554

[jsr13612-bib-0027] Fantini, M. L. , Ferini‐Strambi, L. , & Montplaisir, J. (2005). Idiopathic REM sleep behavior disorder: Toward a better nosologic definition. Neurology, 64, 780–786. 15753409 10.1212/01.WNL.0000152878.79429.00

[jsr13612-bib-0028] Feinstein, M. A. , Sharp, R. R. , Sandness, D. J. , Feemster, J. C. , Junna, M. , Kotagal, S. , Lipford, M. C. , Tippmann‐Peikert, M. , Boeve, B. F. , Silber, M. H. , & St Louis, E. K. (2019). Physician and patient determinants of prognostic counseling in idiopathic REM sleep‐behavior disorder. Sleep Medicine, 62, 80–85. 10.1016/j.sleep.2019.03.010 31581066 PMC6886881

[jsr13612-bib-0029] Fernandez‐Arcos, A. , Iranzo, A. , Serradell, M. , Gaig, C. , & Santamaria, J. (2016). The clinical phenotype of idiopathic rapid eye movement sleep behavior disorder at presentation: A study in 203 consecutive patients. Sleep, 39(1), 121–132. 10.5665/sleep.5332 26940460 PMC4678361

[jsr13612-bib-0030] Fernandez‐Santiago, R. , Iranzo, A. , Gaig, C. , Serradell, M. , Fernandez, M. , Tolosa, E. , Santamaria, J. , & Ezquerra, M. (2016). Absence of LRRK2 mutations in a cohort of patients with idiopathic REM sleep behavior disorder. Neurology, 86(11), 1072–1073. 10.1212/WNL.0000000000002304 26747879

[jsr13612-bib-0031] Ferri, R. , Rundo, F. , Manconi, M. , Plazzi, G. , Bruni, O. , Oldani, A. , Ferini‐Strambi, L. , & Zucconi, M. (2010). Improved computation of the atonia index in normal controls and patients with REM sleep behavior disorder. Sleep Medicine, 11(9), 947–949. 10.1016/j.sleep.2010.06.003 20817596

[jsr13612-bib-0032] Frauscher, B. , Gabelia, D. , Biermayr, M. , Stefani, A. , Hackner, H. , Mitterling, T. , Poewe, W. , & Hogl, B. (2014). Validation of an integrated software for the detection of rapid eye movement sleep behavior disorder. Sleep, 37(10), 1663–1671. 10.5665/sleep.4076 25197814 PMC4173922

[jsr13612-bib-0033] Frauscher, B. , Gschliesser, V. , Brandauer, E. , Ulmer, H. , Peralta, C. M. , Muller, J. , Poewe, W. , & Hogl, B. (2007). Video analysis of motor events in REM sleep behavior disorder. Movement Disorders, 22(10), 1464–1470. 10.1002/mds.21561 17516467

[jsr13612-bib-0034] Frauscher, B. , & Högl, B. (2012). REM sleep behavior disorder: Discovery of RBD, clinical and laboratory diagnosis, and treatment. In A. R. P. Walters , Chokroverty S . (Ed.), Movement disorders in sleep. Springer

[jsr13612-bib-0035] Frauscher, B. , Iranzo, A. , Gaig, C. , Gschliesser, V. , Guaita, M. , Raffelseder, V. , Ehrmann, L. , Sola, N. , Salamero, M. , Tolosa, E. , Poewe, W. , Santamaria, J. , Hogl, B. , & SINBAR (Sleep Innsbruck Barcelona) Group . (2012). Normative EMG values during REM sleep for the diagnosis of REM sleep behavior disorder. Sleep, 35(6), 835–847. 10.5665/sleep.1886 22654203 PMC3353058

[jsr13612-bib-0036] Gan‐Or, Z. , Montplaisir, J. Y. , Ross, J. P. , Poirier, J. , Warby, S. C. , Arnulf, I. , Strong, S. , Dauvilliers, Y. , Leblond, C. S. , Hu, M. T. M. , Hogl, B. , Stefani, A. , Monaca, C. C. , De Cock, V. C. , Boivin, M. , Ferini‐Strambi, L. , Plazzi, G. , Antelmi, E. , Young, P. , … Rouleau, G. A. (2017). The dementia‐associated APOE epsilon4 allele is not associated with rapid eye movement sleep behavior disorder. Neurobiology of Aging, 49, 218 e213–218 e215. 10.1016/j.neurobiolaging.2016.10.002 27814994

[jsr13612-bib-0037] Gilat, M. , Coeytaux Jackson, A. , Marshall, N. S. , Hammond, D. , Mullins, A. E. , Hall, J. M. , Fang, B. A. M. , Yee, B. J. , Wong, K. K. H. , Grunstein, R. R. , & Lewis, S. J. G. (2020). Melatonin for rapid eye movement sleep behavior disorder in Parkinson's disease: A randomised controlled trial. Movement Disorders, 35(2), 344–349. 10.1002/mds.27886 31674060 PMC7027846

[jsr13612-bib-0038] Guilleminault, C. , Raynal, D. , Takahashi, S. , Carskadon, M. , & Dement, W. (1976). Evaluation of short‐term and long‐term treatment of the narcolepsy syndrome with clomipramine hydrochloride. Acta Neurologica Scandinavica, 54(1), 71–87. 936975 10.1111/j.1600-0404.1976.tb07621.x

[jsr13612-bib-0039] Haba‐Rubio, J. , Frauscher, B. , Marques‐Vidal, P. , Toriel, J. , Tobback, N. , Andries, D. , Preisig, M. , Vollenweider, P. , Postuma, R. , & Heinzer, R. (2018). Prevalence and determinants of rapid eye movement sleep behavior disorder in the general population. Sleep, 41(2). 10.1093/sleep/zsx197 29216391

[jsr13612-bib-0040] Hogl, B. , Stefani, A. , & Videnovic, A. (2018). Idiopathic REM sleep behaviour disorder and neurodegeneration – an update. Nature Reviews. Neurology, 14(1), 40–55. 10.1038/nrneurol.2017.157 29170501

[jsr13612-bib-0041] Honeycutt, L. , Montplaisir, J. Y. , Gagnon, J. F. , Ruskey, J. , Pelletier, A. , Gan‐Or, Z. , & Postuma, R. B. (2019). Glucocerebrosidase mutations and phenoconversion of REM sleep behavior disorder to parkinsonism and dementia. Parkinsonism & Related Disorders, 65, 230–233. 10.1016/j.parkreldis.2019.04.016 31076265

[jsr13612-bib-0042] Iranzo, A. (2018). The REM sleep circuit and how its impairment leads to REM sleep behavior disorder. Cell and Tissue Research, 373(1), 245–266. 10.1007/s00441-018-2852-8 29846796

[jsr13612-bib-0043] Iranzo, A. , Borrego, S. , Vilaseca, I. , Marti, C. , Serradell, M. , Sanchez‐Valle, R. , Kovacs, G. G. , Valldeoriola, F. , Gaig, C. , Santamaria, J. , Tolosa, E. , & Gelpi, E. (2018). Alpha‐synuclein aggregates in labial salivary glands of idiopathic rapid eye movement sleep behavior disorder. Sleep, 41(8). 10.1093/sleep/zsy101 29790977

[jsr13612-bib-0044] Iranzo, A. , Fairfoul, G. , Ayudhaya, A. C. N. , Serradell, M. , Gelpi, E. , Vilaseca, I. , Sanchez‐Valle, R. , Gaig, C. , Santamaria, J. , Tolosa, E. , Riha, R. L. , & Green, A. J. E. (2021). Detection of alpha‐synuclein in CSF by RT‐QuIC in patients with isolated rapid‐eye‐movement sleep behaviour disorder: A longitudinal observational study. Lancet Neurology, 20(3), 203–212. 10.1016/S1474-4422(20)30449-X 33609478

[jsr13612-bib-0045] Iranzo, A. , Molinuevo, J. L. , Santamaria, J. , Serradell, M. , Marti, M. J. , Valldeoriola, F. , & Tolosa, E. (2006). Rapid‐eye‐movement sleep behaviour disorder as an early marker for a neurodegenerative disorder: A descriptive study. Lancet Neurology, 5(7), 572–577. 10.1016/S1474-4422(06)70476-8 16781987

[jsr13612-bib-0046] Iranzo, A. , Santamaria, J. , & de Riquer, M. (2004). Sleep and sleep disorders in Don Quixote. Sleep Medicine, 5(1), 97–100. 14725836 10.1016/j.sleep.2003.05.001

[jsr13612-bib-0047] Iranzo, A. , Santamaria, J. , & Tolosa, E. (2016). Idiopathic rapid eye movement sleep behaviour disorder: Diagnosis, management, and the need for neuroprotective interventions. Lancet Neurology, 15(4), 405–419. 10.1016/S1474-4422(16)00057-0 26971662

[jsr13612-bib-0048] Iranzo, A. , Stefani, A. , Serradell, M. , Marti, M. J. , Lomena, F. , Mahlknecht, P. , Stockner, H. , Gaig, C. , Fernandez‐Arcos, A. , Poewe, W. , Tolosa, E. , Hogl, B. , Santamaria, J. , & SINBAR (Sleep Innsbruck Barcelona) Group . (2017). Characterization of patients with longstanding idiopathic REM sleep behavior disorder. Neurology, 89(3), 242–248. 10.1212/WNL.0000000000004121 28615430

[jsr13612-bib-0049] Iranzo, A. , Tolosa, E. , Gelpi, E. , Molinuevo, J. L. , Valldeoriola, F. , Serradell, M. , Sanchez‐Valle, R. , Vilaseca, I. , Lomena, F. , Vilas, D. , Llado, A. , Gaig, C. , & Santamaria, J. (2013). Neurodegenerative disease status and post‐mortem pathology in idiopathic rapid‐eye‐movement sleep behaviour disorder: An observational cohort study. Lancet Neurology, 12(5), 443–453. 10.1016/S1474-4422(13)70056-5 23562390

[jsr13612-bib-0050] Jouvet, M. , & Delorme, F. (1965). Locus coeruleus et sommeil paradoxal. C R Soc Biol, 159, 895–899.

[jsr13612-bib-0051] Krohn, L. , Heilbron, K. , Blauwendraat, C. , Reynolds, R. H. , Yu, E. , Senkevich, K. , Rudakou, U. , Estiar, M. A. , Gustavsson, E. K. , Brolin, K. , Ruskey, J. A. , Freeman, K. , Asayesh, F. , Chia, R. , Arnulf, I. , Hu, M. T. M. , Montplaisir, J. Y. , Gagnon, J.‐F. , Desautels, A. , … Gan‐Or, Z. (2021). Genome‐wide association study of REM sleep behavior disorder identifies novel loci with distinct polygenic and brain expression effects. *medRxiv*, 2021.2009.2008.21254232. doi:10.1101/2021.09.08.21254232 PMC9722930 36470867

[jsr13612-bib-0052] Krohn, L. , Wu, R. Y. J. , Heilbron, K. , Ruskey, J. A. , Laurent, S. B. , Blauwendraat, C. , Alam, A. , Arnulf, I. , Hu, M. T. M. , Dauvilliers, Y. , Hogl, B. , Toft, M. , Bjornara, K. A. , Stefani, A. , Holzknecht, E. , Monaca, C. C. , Abril, B. , Plazzi, G. , Antelmi, E. , … Gan‐Or, Z. (2020). Fine‐mapping of SNCA in rapid eye movement sleep behavior disorder and overt synucleinopathies. Annals of Neurology, 87(4), 584–598. 10.1002/ana.25687 31976583 PMC8025046

[jsr13612-bib-0053] Lasègue, C. (1881). Le délire alcoolique n'est pas un délire mais un rêve. Archives Géneral Médecine, 88, 513–536.

[jsr13612-bib-0054] Mahale, R. R. , Yadav, R. , & Pal, P. K. (2016). Rapid eye movement sleep behaviour disorder in women with Parkinson's disease is an underdiagnosed entity. Journal of Clinical Neuroscience, 28, 43–46. 10.1016/j.jocn.2015.08.046 26765761

[jsr13612-bib-0055] McCarter, S. J. , Sandness, D. J. , McCarter, A. R. , Feemster, J. C. , Teigen, L. N. , Timm, P. C. , Boeve, B. F. , Silber, M. H. , & St Louis, E. K. (2019). REM sleep muscle activity in idiopathic REM sleep behavior disorder predicts phenoconversion. Neurology, 93(12), e1171–e1179. 10.1212/WNL.0000000000008127 31420463 PMC6808528

[jsr13612-bib-0056] Miglis, M. G. , Adler, C. H. , Antelmi, E. , Arnaldi, D. , Baldelli, L. , Boeve, B. F. , Cesari, M. , Dall'Antonia, I. , Diederich, N. J. , Doppler, K. , Dusek, P. , Ferri, R. , Gagnon, J. F. , Gan‐Or, Z. , Hermann, W. , Hogl, B. , Hu, M. T. , Iranzo, A. , Janzen, A. , … Oertel, W. H. (2021). Biomarkers of conversion to alpha‐synucleinopathy in isolated rapid‐eye‐movement sleep behaviour disorder. Lancet Neurology, 20(8), 671–684. 10.1016/S1474-4422(21)00176-9 34302789 PMC8600613

[jsr13612-bib-0057] Mollenhauer, B. , Caspell‐Garcia, C. J. , Coffey, C. S. , Taylor, P. , Singleton, A. , Shaw, L. M. , Trojanowski, J. Q. , Frasier, M. , Simuni, T. , Iranzo, A. , Oertel, W. , Siderowf, A. , Weintraub, D. , Seibyl, J. , Toga, A. W. , Tanner, C. M. , Kieburtz, K. , Chahine, L. M. , Marek, K. , … for the PPMI study . (2019). Longitudinal analyses of cerebrospinal fluid alpha‐synuclein in prodromal and early Parkinson's disease. Movement Disorders, 34(9), 1354–1364. 10.1002/mds.27806 31361367 PMC7098385

[jsr13612-bib-0058] Mollenhauer, B. , Dakna, M. , Kruse, N. , Galasko, D. , Foroud, T. , Zetterberg, H. , Schade, S. , Gera, R. G. , Wang, W. , Gao, F. , Frasier, M. , Chahine, L. M. , Coffey, C. S. , Singleton, A. B. , Simuni, T. , Weintraub, D. , Seibyl, J. , Toga, A. W. , Tanner, C. M. , … Graham, D. (2020). Validation of serum neurofilament light chain as a biomarker of Parkinson's disease progression. Movement Disorders, 35(11), 1999–2008. 10.1002/mds.28206 32798333 PMC8017468

[jsr13612-bib-0059] Mouret, J. (1975). Differences in sleep in patients with Parkinson's disease. Electroencephalography and Clinical Neurophysiology, 38(6), 653–657. 50193 10.1016/0013-4694(75)90168-6

[jsr13612-bib-0060] Mufti, K. , Rudakou, U. , Yu, E. , Krohn, L. , Ruskey, J. A. , Asayesh, F. , Laurent, S. B. , Spiegelman, D. , Arnulf, I. , Hu, M. T. M. , Montplaisir, J. Y. , Gagnon, J. F. , Desautels, A. , Dauvilliers, Y. , Gigli, G. L. , Valente, M. , Janes, F. , Hogl, B. , Stefani, A. , … Gan‐Or, Z. (2021a). Comprehensive analysis of familial parkinsonism genes in rapid‐eye‐movement sleep behavior disorder. Movement Disorders, 36(1), 235–240. 10.1002/mds.28318 33001463

[jsr13612-bib-0061] Mufti, K. , Yu, E. , Rudakou, U. , Krohn, L. , Ruskey, J. A. , Asayesh, F. , Laurent, S. B. , Spiegelman, D. , Arnulf, I. , Hu, M. T. M. , Montplaisir, J. Y. , Gagnon, J. F. , Desautels, A. , Dauvilliers, Y. , Gigli, G. L. , Valente, M. , Janes, F. , Bernardini, A. , Hogl, B. , … Gan‐Or, Z. (2021b). Novel associations of BST1 and LAMP3 with REM sleep behavior disorder. Neurology, 96(10), e1402–e1412. 10.1212/WNL.0000000000011464 33397775 PMC8055320

[jsr13612-bib-0062] Nalls, M. A. , Blauwendraat, C. , Vallerga, C. L. , Heilbron, K. , Bandres‐Ciga, S. , Chang, D. , Tan, M. , Kia, D. A. , Noyce, A. J. , Xue, A. , Bras, J. , Young, E. , von Coelln, R. , Simon‐Sanchez, J. , Schulte, C. , Sharma, M. , Krohn, L. , Pihlstrom, L. , Siitonen, A. , … International Parkinson's Disease Genomics Consortium . (2019). Identification of novel risk loci, causal insights, and heritable risk for Parkinson's disease: A meta‐analysis of genome‐wide association studies. Lancet Neurology, 18(12), 1091–1102. 10.1016/S1474-4422(19)30320-5 31701892 PMC8422160

[jsr13612-bib-0063] Nepozitek, J. , Dostalova, S. , Dusek, P. , Kemlink, D. , Prihodova, I. , Ibarburu Lorenzo, Y. L. V. , Friedrich, L. , Bezdicek, O. , Nikolai, T. , Perinova, P. , Dall'Antonia, I. , Dusek, P. , Ruml, M. , Ruzicka, E. , & Sonka, K. (2019). Simultaneous tonic and phasic REM sleep without atonia best predicts early phenoconversion to neurodegenerative disease in idiopathic REM sleep behavior disorder. Sleep, 42(9). 10.1093/sleep/zsz132 31194249

[jsr13612-bib-0064] Niiyama, Y. , Shimizu, T. , Abe, M. , & Hishikawa, Y. (1993). Cortical reactivity in REM sleep with tonic mentalis EMG activity induced by clomipramine: An evaluation by slow vertex response. Electroencephalography and Clinical Neurophysiology, 86(4), 247–251. 10.1016/0013-4694(93)90105-5 7682927

[jsr13612-bib-0065] Pline l'Ancien (1961). Livre X (Des Animaux ailés). In Saint‐Denis, E. D. (Eds.), Histoire naturelle (Vol. XCVIII, p. 248). Les belles lettres.

[jsr13612-bib-0066] Postuma, R. B. , Iranzo, A. , Hu, M. , Hogl, B. , Boeve, B. F. , Manni, R. , Oertel, W. H. , Arnulf, I. , Ferini‐Strambi, L. , Puligheddu, M. , Antelmi, E. , Cochen De Cock, V. , Arnaldi, D. , Mollenhauer, B. , Videnovic, A. , Sonka, K. , Jung, K. Y. , Kunz, D. , Dauvilliers, Y. , … Pelletier, A. (2019). Risk and predictors of dementia and parkinsonism in idiopathic REM sleep behaviour disorder: A multicentre study. Brain, 142(3), 744–759. 10.1093/brain/awz030 30789229 PMC6391615

[jsr13612-bib-0067] Postuma, R. B. , Montplaisir, J. Y. , Pelletier, A. , Dauvilliers, Y. , Oertel, W. , Iranzo, A. , Ferini‐Strambi, L. , Arnulf, I. , Hogl, B. , Manni, R. , Miyamoto, T. , Mayer, G. , Stiasny‐Kolster, K. , Puligheddu, M. , Ju, Y. , Jennum, P. , Sonka, K. , Santamaria, J. , Fantini, M. L. , … Wolfson, C. (2012). Environmental risk factors for REM sleep behavior disorder: A multicenter case‐control study. Neurology, 79(5), 428–434. 10.1212/WNL.0b013e31825dd383 22744670 PMC3405255

[jsr13612-bib-0068] Pujol, M. , Pujol, J. , Alonso, T. , Fuentes, A. , Pallerola, M. , Freixenet, J. , Barbe, F. , Salamero, M. , Santamaria, J. , & Iranzo, A. (2017). Idiopathic REM sleep behavior disorder in the elderly Spanish community: A primary care center study with a two‐stage design using video‐polysomnography. Sleep Medicine, 40, 116–121. 10.1016/j.sleep.2017.07.021 29042180

[jsr13612-bib-0069] Rekling, J. C. , Funk, G. D. , Bayliss, D. A. , Dong, X. W. , & Feldman, J. L. (2000). Synaptic control of motoneuronal excitability. Physiological Reviews, 80(2), 767–852. 10.1152/physrev.2000.80.2.767 10747207 PMC4764886

[jsr13612-bib-0070] Salva, M. A. , & Guilleminault, C. (1986). Olivopontocerebellar degeneration, abnormal sleep, and REM sleep without atonia. Neurology, 36(4), 576–577. 10.1212/wnl.36.4.576 3960336

[jsr13612-bib-0071] Sapin, E. , Lapray, D. , Berod, A. , Goutagny, R. , Leger, L. , Ravassard, P. , Clement, O. , Hanriot, L. , Fort, P. , & Luppi, P. H. (2009). Localization of the brainstem GABAergic neurons controlling paradoxical (REM) sleep. PLoS One, 4(1), e4272. 10.1371/journal.pone.0004272 19169414 PMC2629845

[jsr13612-bib-0072] Sastre, J. P. , & Jouvet, M. (1979). Oneiric behavior in cats. Physiology and Behavior, 22(5), 979–989. 10.1016/0031-9384(79)90344-5 228328

[jsr13612-bib-0073] Schenck, C. H. , Bundlie, S. R. , Ettinger, M. G. , & Mahowald, M. W. (1986). Chronic behavioral disorders of human REM sleep: A new category of parasomnia. Sleep, 9(2), 293–308. 10.1093/sleep/9.2.293 3505730

[jsr13612-bib-0074] Schenck, C. H. , Bundlie, S. R. , & Mahowald, M. W. (1996). Delayed emergence of a parkinsonian disorder in 38% of 29 older men initially diagnosed with idiopathic rapid eye movement sleep behaviour disorder. Neurology, 46, 388–393. 8614500 10.1212/wnl.46.2.388

[jsr13612-bib-0075] Schenck, C. H. , Högl, B. , & Videnovic, A. (2019). Rapid‐Eye‐Movement Sleep Behavior Disorder. Springer International Publishing AG, part of Springer Nature, 2019, 215–222. 10.1007/978-3-319-90152-7_1

[jsr13612-bib-0076] Schenck, C. H. , Montplaisir, J. Y. , Frauscher, B. , Hogl, B. , Gagnon, J. F. , Postuma, R. , Sonka, K. , Jennum, P. , Partinen, M. , Arnulf, I. , Cochen de Cock, V. , Dauvilliers, Y. , Luppi, P. H. , Heidbreder, A. , Mayer, G. , Sixel‐Doring, F. , Trenkwalder, C. , Unger, M. , Young, P. , … Oertel, W. (2013). Rapid eye movement sleep behavior disorder: Devising controlled active treatment studies for symptomatic and neuroprotective therapy – a consensus statement from the international rapid eye movement sleep behavior disorder study group. Sleep Medicine, 14(8), 795–806. 10.1016/j.sleep.2013.02.016 23886593 PMC8783206

[jsr13612-bib-0077] Shahnawaz, M. , Mukherjee, A. , Pritzkow, S. , Mendez, N. , Rabadia, P. , Liu, X. , Hu, B. , Schmeichel, A. , Singer, W. , Wu, G. , Tsai, A. L. , Shirani, H. , Nilsson, K. P. R. , Low, P. A. , & Soto, C. (2020). Discriminating alpha‐synuclein strains in Parkinson's disease and multiple system atrophy. Nature, 578(7794), 273–277. 10.1038/s41586-020-1984-7 32025029 PMC7066875

[jsr13612-bib-0078] Shahnawaz, M. , Tokuda, T. , Waragai, M. , Mendez, N. , Ishii, R. , Trenkwalder, C. , Mollenhauer, B. , & Soto, C. (2017). Development of a biochemical diagnosis of Parkinson disease by detection of alpha‐synuclein misfolded aggregates in cerebrospinal fluid. JAMA Neurology, 74(2), 163–172. 10.1001/jamaneurol.2016.4547 27918765

[jsr13612-bib-0079] Shimizu, T. , Inami, Y. , Sugita, Y. , Iijima, S. , Teshima, Y. , Matsuo, R. , Yasoshima, A. , Egawa, I. , Okawa, M. , Tashiro, T. (1990). REM sleep without muscle atonia (stage 1‐REM) and its relation to delirious behavior during sleep in patients with degenerative diseases involving the brain stem. The Japanese Journal of Psychiatry and Neurology, 44(4), 681–692. 10.1111/j.1440-1819.1990.tb01645.x 2096238

[jsr13612-bib-0080] Shin, C. , Park, H. , Lee, W. W. , Kim, H. J. , Kim, H. J. , & Jeon, B. (2019). Clonazepam for probable REM sleep behavior disorder in Parkinson's disease: A randomized placebo‐controlled trial. Journal of the Neurological Sciences, 401, 81–86. 10.1016/j.jns.2019.04.029 31035190

[jsr13612-bib-0081] Sosero, Y. L. , Yu, E. , Estiar, M. A. , Krohn, L. , Mufti, K. , Rudakou, U. , Ruskey, J. A. , Asayesh, F. , Laurent, S. B. , Spiegelman, D. , Trempe, J. F. , Quinnell, T. G. , Oscroft, N. , Arnulf, I. , Montplaisir, J. Y. , Gagnon, J. F. , Desautels, A. , Dauvilliers, Y. , Gigli, G. L. , … Gan‐Or, Z. (2022). Rare PSAP variants and possible interaction with GBA in REM sleep behavior disorder. Journal of Parkinson's Disease, 12(1), 333–340. 10.3233/JPD-212867 34690151

[jsr13612-bib-0082] Sprenger, F. S. , Stefanova, N. , Gelpi, E. , Seppi, K. , Navarro‐Otano, J. , Offner, F. , Vilas, D. , Valldeoriola, F. , Pont‐Sunyer, C. , Aldecoa, I. , Gaig, C. , Gines, A. , Cuatrecasas, M. , Hogl, B. , Frauscher, B. , Iranzo, A. , Wenning, G. K. , Vogel, W. , Tolosa, E. , & Poewe, W. (2015). Enteric nervous system alpha‐synuclein immunoreactivity in idiopathic REM sleep behavior disorder. Neurology, 85(20), 1761–1768. 10.1212/WNL.0000000000002126 26475692 PMC4653104

[jsr13612-bib-0083] St Louis, E. K. , & Boeve, B. F. (2017). REM sleep behavior disorder: Diagnosis, clinical implications, and future directions. Mayo Clinic Proceedings, 92(11), 1723–1736. 10.1016/j.mayocp.2017.09.007 29101940 PMC6095693

[jsr13612-bib-0084] Stefani, A. , Iranzo, A. , Holzknecht, E. , Perra, D. , Bongianni, M. , Gaig, C. , Heim, B. , Serradell, M. , Sacchetto, L. , Garrido, A. , Capaldi, S. , Sánchez‐Gómez, A. , Cecchini, MP. , Mariotto, S. , Ferrari, S. , Fiorini, M. , Schmutzhard, J. , Cocchiara, P. , Vilaseca, I. , … SINBAR (Sleep Innsbruck Barcelona) group . (2021). Alpha‐synuclein seeds in olfactory mucosa of patients with isolated REM sleep behaviour disorder. Brain, 144(4):1118–1126. 10.1093/brain/awab005. 33855335

[jsr13612-bib-0085] Tachibana, N. (2009). Historical overview of REM sleep behavior disorder in relation to its pathophysiology. Brain and Nerve, 61(5), 558–568. 19514516

[jsr13612-bib-0086] Tachibana, N. , Sugita, Y. , Terashima, K. , Teshima, Y. , Shimizu, T. , & Hishikawa, Y. (1991). Polysomnographic characteristics of healthy elderly subjects with somnambulism‐like behaviors. Biological Psychiatry, 30(1), 4–14. 10.1016/0006-3223(91)90065-T 1892961

[jsr13612-bib-0087] Tatman, J. , & Sind, J. (1996). REM behavior disorder manifests differently in women and men. Sleep Research, 25, 380.

[jsr13612-bib-0088] Teigen, L. N. , Sharp, R. R. , Hirsch, J. R. , Campbell, E. , Timm, P. C. , Sandness, D. J. , Feemster, J. C. , Gossard, T. R. , Faber, S. M. , Steele, T. A. , Rivera, S. , Junna, M. R. , Lipford, M. C. , Tippmann‐Peikert, M. , Kotagal, S. , Ju, Y. E. , Howell, M. , Schenck, C. H. , Videnovic, A. , … St Louis, E. K. (2021). Specialist approaches to prognostic counseling in isolated REM sleep behavior disorder. Sleep Medicine, 79, 107–112. 10.1016/j.sleep.2020.12.014 33486257 PMC10075000

[jsr13612-bib-0089] Traczynska‐Kubin, D. , Atzef, E. , & Petre‐Quadens, O. (1969). Sleep in parkinsonism. Acta Neurologica et Psychiatrica Belgica, 69(9), 727–733. 4327788

[jsr13612-bib-0090] Uchida, S. , Soya, S. , Saito, Y. C. , Hirano, A. , Koga, K. , Tsuda, M. , Abe, M. , Sakimura, K. , & Sakurai, T. (2021). A discrete glycinergic neuronal population in the ventromedial medulla that induces muscle atonia during REM sleep and cataplexy in mice. The Journal of Neuroscience, 41(7), 1582–1596. 10.1523/JNEUROSCI.0688-20.2020 33372061 PMC7896014

[jsr13612-bib-0091] Uchiyama, M. , Isse, K. , Tanaka, K. , Yokota, N. , Hamamoto, M. , Aida, S. , Ito, Y. , Yoshimura, M. , & Okawa, M. (1995). Incidental Lewy body disease in a patient with REM sleep behavior disorder. Neurology, 45, 709–712. 7723959 10.1212/wnl.45.4.709

[jsr13612-bib-0092] Valencia Garcia, S. , Brischoux, F. , Clement, O. , Libourel, P. A. , Arthaud, S. , Lazarus, M. , Luppi, P. H. , & Fort, P. (2018). Ventromedial medulla inhibitory neuron inactivation induces REM sleep without atonia and REM sleep behavior disorder. Nature Communications, 9(1), 504. 10.1038/s41467-017-02761-0 PMC5799338 29402935

[jsr13612-bib-0093] Valencia Garcia, S. , Libourel, P. A. , Lazarus, M. , Grassi, D. , Luppi, P. H. , & Fort, P. (2017). Genetic inactivation of glutamate neurons in the rat sublaterodorsal tegmental nucleus recapitulates REM sleep behaviour disorder. Brain, 140(2), 414–428. 10.1093/brain/aww310 28007991

[jsr13612-bib-0094] Videnovic, A. , Ju, Y. S. , Arnulf, I. , Cochen‐De Cock, V. , Hogl, B. , Kunz, D. , Provini, F. , Ratti, P. L. , Schiess, M. C. , Schenck, C. H. , Trenkwalder, C. , & Treatment and Trials Working Group of the International RBD Study Group . (2020). Clinical trials in REM sleep behavioural disorder: Challenges and opportunities. Journal of Neurology, Neurosurgery, and Psychiatry, 91(7), 740–749. 10.1136/jnnp-2020-322875 32404379 PMC7735522

[jsr13612-bib-0095] Vilas, D. , Iranzo, A. , Tolosa, E. , Aldecoa, I. , Berenguer, J. , Vilaseca, I. , Marti, C. , Serradell, M. , Lomena, F. , Alos, L. , Gaig, C. , Santamaria, J. , & Gelpi, E. (2016). Assessment of alpha‐synuclein in submandibular glands of patients with idiopathic rapid‐eye‐movement sleep behaviour disorder: A case‐control study. Lancet Neurology, 15(7), 708–718. 10.1016/S1474-4422(16)00080-6 27039162

[jsr13612-bib-0096] Wang, Z. , Becker, K. , Donadio, V. , Siedlak, S. , Yuan, J. , Rezaee, M. , Incensi, A. , Kuzkina, A. , Orru, C. D. , Tatsuoka, C. , Liguori, R. , Gunzler, S. A. , Caughey, B. , Jimenez‐Capdeville, M. E. , Zhu, X. , Doppler, K. , Cui, L. , Chen, S. G. , Ma, J. , & Zou, W. Q. (2020). Skin alpha‐synuclein aggregation seeding activity as a novel biomarker for Parkinson disease. JAMA Neurology, 78(1), 30‐40. 10.1001/jamaneurol.2020.3311 PMC7522783 32986090

[jsr13612-bib-0097] Waser, M. , Stefani, A. , Holzknecht, E. , Kohn, B. , Hackner, H. , Brandauer, E. , Bergmann, M. , Taupe, P. , Gall, M. , Garn, H. , & Hogl, B. (2020). Automated 3D video analysis of lower limb movements during REM sleep: A new diagnostic tool for isolated REM sleep behavior disorder. Sleep, 43(11). 10.1093/sleep/zsaa100 PMC7658637 32573731

[jsr13612-bib-0098] Zhou, J. , Zhang, J. , Li, Y. , Du, L. , Li, Z. , Lei, F. , Wing, Y. K. , Kushida, C. A. , Zhou, D. , & Tang, X. (2015). Gender differences in REM sleep behavior disorder: A clinical and polysomnographic study in China. Sleep Medicine, 16(3), 414–418. 10.1016/j.sleep.2014.10.020 25660814

